# Why Engagement Matters to Gerontological Student Learning

**DOI:** 10.1093/geroni/igaa057.1771

**Published:** 2020-12-16

**Authors:** Christine Fruhauf, Allyson Brothers, Nicole Ehrhart, Blake Naughton, Sue Schneider

**Affiliations:** 1 Colorado State University, Fort Collins, Colorado, United States; 2 Larimer County Extension, Fort Collins, Colorado, United States

## Abstract

Learning opportunities beyond the classroom create lasting and positive effects on students’ academic and professional growth. When faculty members participate in engaged scholarship in their local communities, out-of-classroom learning opportunities for students likewise expand. In the context of aging, the array of opportunities for students to engage with community partners is vast, from interactive course assignments to research opportunities, clinical experience, local policy efforts, and more. This paper builds on previous theoretical and empirical work from scholars (including AGHE and GSA Fellows) in the science of engagement, and integrates case examples of campus-community relationships from our collective years in the academy. Examples will showcase how engaged teaching, engaged research, and engaged service collectively informs and enriches our students’ experiences. In the spirit of the 2020 conference theme, we will discuss opportunities for university-community engagement as a way to strengthen and inspire current and future gerontological pedagogy.

